# Structure Elucidation and Immunomodulatory Activity of A Beta Glucan from the Fruiting Bodies of *Ganoderma sinense*

**DOI:** 10.1371/journal.pone.0100380

**Published:** 2014-07-11

**Authors:** Xiao-Qiang Han, Gar-Lee Yue, Rui-Qi Yue, Cai-Xia Dong, Chung-Lap Chan, Chun-Hay Ko, Wing-Shing Cheung, Ke-Wang Luo, Hui Dai, Chun-Kwok Wong, Ping-Chung Leung, Quan-Bin Han

**Affiliations:** 1 State Key Laboratory of Phytochemistry and Plant Resources in West China, Institute of Chinese Medicine, The Chinese University of Hong Kong, Shatin, Hong Kong SAR, China; 2 Shenzhen Research Institute, The Chinese University of Hong Kong, Shenzhen, Guangdong, China; 3 School of Chinese Medicine, Hong Kong Baptist University, Kowloon, Hong Kong SAR, China; 4 Department of Immunology, School of Basic Medical Sciences, Peking University Health Science Center, Beijing, China; Macau University of Science and Technology, Macao

## Abstract

A polysaccharide named GSP-2 with a molecular size of 32 kDa was isolated from the fruiting bodies of *Ganoderma sinense*. Its structure was well elucidated, by a combined utilization of chemical and spectroscopic techniques, to be a β-glucan with a backbone of (1→4)– and (1→6)–Glc*p*, bearing *terminal-* and (1→3)–Glc*p* side-chains at *O*-3 position of (1→6)–Glc*p*. Immunological assay exhibited that GSP-2 significantly induced the proliferation of BALB/c mice splenocytes with target on only B cells, and enhanced the production of several cytokines in human peripheral blood mononuclear cells and derived dendritic cells. Besides, the fluorescent labeled GSP-2 was phagocytosed by the RAW 264.7 cells and induced the nitric oxide secretion from the cells.

## Introduction

Lingzhi is a well-known anticancer fungus. Although it is the general name of *Ganoderma* species, the name Lingzhi usually means a single species *G. lucidum*, which is one of the most studied mushrooms in the world [1]. Its anti-cancer effects are associated with not only the cytotoxic triterpenoids, but also the immunomodulating polysaccharides via the inhibition of DNA polymerase and post-translational modification of the Ras oncoprotein, or the stimulation of cytokine production [2]–[5].

While *Ganoderma sinense* is also called the same name “Lingzhi” like *G. lucidum*, as recorded in Chinese Pharmacopoeia 2010, it is only distributed in China and contains little ganoderic acid like triterpenoids [6]–[7]. Both official Lingzhi species are rich in polysaccharides and possess multiple biological activities [1], such as antimicrobials [6]–[7], immunomodulation [8], and antitumor effect [9]. Since *G. sinense* is traditionally used in the form of decoction, the water-soluble polysaccharides are also considered its major active ingredients, having immune-balancing [10], antioxidant [11]–[12] and antitumor activities [13]. It was reported that the protein-bound polysaccharide from the fruiting bodies of *G. Sinense* could significantly induce the proliferation of human peripheral blood mononuclear cells (PBMCs), in the meanwhile enhance the secretion of tumor necrosis factor-α, interleukin (IL)-10 and IL-12 and transforming growth factor-β from PBMCs and enhance the population of CD14^+^ cell [13]. Unfortunately, few polysaccharides have been purified from *G. sinense* and little is known about their chemistry and bioactivity [1].

In our previous study, two purified polysaccharides GSP-6B and GSP-4 were reported from the fruiting bodies of *G. sinense* [14]–[15]. Their chemical structures were partially elucidated to be a hyperbranched β-glucan and a heteropolysaccharide, respectively. Additionally, both polysaccharides exhibited immunomodulating effects *in vitro* without cytotoxicity. However, they might not fully explain the chemistry and immunomodulating effects of *G. sinense* because of their low yield. In this study, we report on the structure elucidation and immunomodulatory activities of another new polysaccharide (GSP-2) which is isolated as a major polysaccharide fraction from the fruiting bodies of *G. sinense.*

## Experimental Section

### Materials and Chemicals

Dried fruiting bodies of *G. sinense* were purchased from herbal store in Hong Kong and authenticated by Professor Zhu-Liang Yang at Kunming Institute of Botany, Chinese Academy of Sciences. The voucher specimen was deposited at the Institute of Chinese Medicine, the Chinese University of Hong Kong, with the voucher specimen number 2010-3271. DEAE Sepharose CL-6B was purchased from GE Healthcare (UK). Reference monosaccharides, T-series dextrans, trifluoroacetic acid (TFA), dimethyl sulfoxide (DMSO), Griess Reagent and lipopolysaccharide (LPS) were purchased from Sigma (St. Louis, MO, USA). RPMI 1640 medium, fetal bovine serum (FBS) and penicillin/streptomycin (100 U/ml) were obtained from Invitrogen (NY, USA). The Raw 264.7 cells were purchased from ATCC, the Global Bioresource Center (Manassas, VA, USA). The fresh human buffy coats were supplied by Hong Kong Red Cross Blood Transfusion Service. The use of human buffy coat for experiment was approved by The Joint Chinese University of Hong Kong-New Territories East Cluster Clinical Research Ethics Committee. The IL-1β, IL-2, IL-4, IL-10, IL-12, IFN-γ, TNF-α ELISA test kits were purchased from BD Pharmingen (CA, USA). GM-CSF was the product of Norvatis (Switzerland).

### General methods

The UV-vis spectra were tested on a Beckman DU650 ultraviolet and visible spectrophotometer. Gas chromatography mass spectrometry (GC-MS) tests for sugar composition analysis were performed on a Shimadzu QP-2010 instrument equipped with a DB-5 column (30 m×0.25 mm×0.25 µm) and a quadrupole rods mass detector (225 °C), the column temperature was increased from 140 °C to 225 °C in a rate of 2 °C/min then hold on 5 min. Gas GC-MS tests for methylation analysis were measured with a DB-5 column (30 m×0.25 mm×0.25 µm), and at temperatures programmed from 170–225°C at 2°C/min and then hold on 10 min. The FT-IR spectra (KBr pellets) were recorded on SPECORD in a range of 400–4000 cm^−1^. The absolute configuration test of the sugar residues were conducted under the method of Gerwig, Kamerling and Vliegenthart [16]–[17].

### Extraction and purification of the polysaccharide

The extraction and preparation of crude polysaccharide has been reported before [14]–[15], after that one in five of the crude polysaccharides (1.0 g) dissolved in 50 mL water was loaded on a DEAE Sepharose CL-6B column (5.0×70.0 cm), and eluted with a 6-step gradient of distilled water, and sodium hydroxide. Guided by the colorimetric total carbohydrate test using the phenol-sulfuric acid method, after all the crude polysaccharide was separated by the DEAE Sepharose CL-6B column, the 0.1 M sodium chloride eluting fractions were combined, dialyzed, lyophilized, and futher purified by a series of Sepharcyl S-300 and Sepharcyl S-400 gel-permeation chromatographic process (2.6 cm×60 cm), eluted with water to afford a purified polysaccharide (GSP-2, 656 mg).

### Determination of homogeneity and molecular weight

The homogeneity and molecular weight of GSP-2 were determined on an Agilent 1100 system equipped with an ELSD detector. Samples (2 mg/mL, 10 µL) were applied to TSK GMPW_XL_ gel filtration columns (7.8×300 mm×2) and eluted with 20 mM CH_3_COONH_4_ at 0.6 mL/min with column temperature maintained at 40°C. Commercially available T-series dextrans (MW 2000, 670, 410, 270, 150, 80, 50, 12, 5 and 1 kD) were used as standard molecular markers.

### Monosaccharide composition analysis

The identification and quantification of the monosaccharides of GSP-2 was achieved by GC-MS analysis. GSP-2 (10 mg) was hydrolyzed with 2 M trifluoroacetic acid (TFA) at 100 °C for 3 h [18]. The monosaccharides were conventionally converted into their completely acetylated derivation and analyzed by GC-MS according to the method of Lawrence and Lyengar [19].

### Methylation analysis

GSP-2 (10 mg) was methylated three times according to the method reported by Needs [20]. Complete methylation was confirmed by the disappearance of the OH band (3200–3700 cm^-1^) in the IR spectrum. The methylated products were hydrolyzed, reduced and acetylated [21]. The partially methylated alditol acetates were analyzed by GC-MS.

### Partial acid hydrolysis

50 mg of GSP-2 was partially hydrolyzed with 0.1 M TFA at 60 °C for 10 h [18]. The resulting hydrolysate was then dialyzed against distilled water for 24 h by a dialysis bag with 7000 Da cut-off. The non-dialysate was collected and coded as GSP-2-P for sugar composition and methylation analysis under the same protocol of the native polysaccharide GSP-2.

### NMR studies

GSP-2 (25 mg) was dried in vacuum under the presence of P_2_O_5_ for 72 h, and then exchanged with deuterium by lyophilizing with D_2_O for three times. The deuterium exchanged polysaccharide was put in a 5-mm NMR tube and dissolved in 1.0 mL 99.96% D_2_O. All 1D and 2D NMR spectrums were acquired at 298 K on a Bruker Avance 500 MHz NMR spectrometer. Tetramethylsilane (TMS) was used as external standard for the ^13^C NMR spectrum, and D_2_O was used as internal standard for ^1^H NMR spectrum.

### Proliferation of mouse splenocytes, splenic T, B cells after GSP-2 treatment

Female BALB/c mice, 8–10 weeks old (18–22 g), were purchased from the Experimental Animal Division of Peking University Health Sciences Center, Beijing, China. All animals were maintained at the specific pathogen free (SPF) laboratory of Experimental Animal Division of Peking University Health Science Center,with stable temperature, food and water supplying. All animal experiments were carried out with the permission of Beijing Experimental Animal Management Authority, Beijing, China, at the animal facilities of this department. The mice were sacrificed by the method of cervical dislocation,all efforts were made to minimize suffering,after that the spleens were collected. Spleens were gently smashed by pressing with the flat surface of a syringe plunger against stainless steel sieve (200 mesh). Red blood cells were lysed by brief treatment with distilled water. The splenocytes were washed twice and then resuspended in complete RPMI 1640 medium supplemented with 10% (v/v) FBS, penicillin/streptomycin (100 U/mL), L-glutamine (2 mM).

Freshly prepared splenocytes resuspended in RPMI 1640 medium at 10^7^ cells/mL were plated into 100 mm tissue culture dishes and incubated for 4 h at 37°C in a CO_2_ incubator. The non-adherent cells were collected, washed twice in PBS, and then incubated with MACS magnetic microbeads coated with rat anti-mouse CD19 mAb (Myltenyi Biotec, Italy) at a density of 10 µL antibody solution/10^7^ splenocytes for 30 min at 4°C. The labeled cells were washed twice with PBS and then applied to a MACS separation column. The effluent was collected as non-B cells. After further washes, the column was removed from the magnet separator and the B cells were flushed out of the column using a plunger. To increase the purity of T cells in the non-B cells, the non-B cell fraction was reapplied to a separation column and the effluent was collected as T cells. The isolated B cells were reacted with FITC-anti mouse CD19 antibody and T cells were reacted with FITC-anti mouse CD3 antibody for 30 min at 4°C. The purities of B cells and T cells were greater than 95% as evaluated by flow cytometry analysis.

Freshly prepared mouse splenocytes (4×10^5^ cells/well), T cells (2×10^5^ cells/well), and B cells (2×10^5^ cells/well), were cultured in flat-bottomed 96-well plates (Nunc, Denmark) in a volume of 200 µl/well. Different concentrations of GSP-2 (10, 30, 100 µg/mL) or Dextran (50 µg/mL) or LPS (2 µg/mL) or ConA (2 µg/mL) were added to the wells. The cells were incubated at 37°C and 5% CO_2_ for 48 h. In the last 8 h of incubation, [^3^H] thymidine ([^3^H]TdR, 0.2 µCi/well) was added into each well. The cells were then harvested, using a 96-well plate harvester (Tomtec, USA), onto fiberglass filters and radioactivity on the filtermatt was counted in a MicroBeta Trilux LSC counter (EG&G Wallac, USA). For inhibition experiments Polymixin B (PMB) (10 ug/ml) was added into the culture system.

### Nitric oxide production and phagocytosis of GSP-2 on macrophages

Macrophages RAW264.7 (4×10^5^ cell/well) were seeded in 24-well plate overnight. Various concentration of GSP-2 (0, 100, 200, 400, 800 µg/mL) or LPS (10 µg/mL) were added and incubated for 24 h. Culture supernatant was added to Griess Reagent in the ratio of 1∶1 in a 96-well plate and the plate was incubated in darkness for 10 min. The plates were then read at a wavelength of 540 nm spectrophotometrically. Nitrite standard curve was plotted with standard NaNO_2_ solution with Griess treatment to determine the concentration of nitrite in culture supernatant samples.

In order to detect the phagocytosis of polysaccharide in macrophages, the polysaccharide GSP-2 was labeled with Alexa Fluor 488 succinimidyl ester (Molecular Probes, Eugene, OR) according to the reported protocol [22]. Briefly, a diaminopropane (DAP) moiety was added to the reducing terminus of the polysaccharides chain of GSP-2 by sodium borohydride reduction to produce a DAP-GSP-2. Then the amine reactive AlexaFluor 488 succinimidyl ester was attached to the DAP moiety located at the reducing terminus of GSP-2 to produce the fluorescent labeled polysaccharide GSP-2. For experiments, the RAW 264.7 cells (5×10^4^ cells/well) were seeded at 24-well culture plates and incubated overnight. The cells were treated with 10 µg (in 1 mL PBS) labeled GSP-2 for 24 h. After incubation, the cells were collected, washed with PBS twice and resuspended in PBS. The fluorescence of the samples was detected by flow cytometry (Becton Dickinson FACSCanto II). The cells were also visualized and imaged under inverted fluorescent microscope (Olympus IX-71).

### Cytokine production of human peripheral blood mononucelar cells and dendritic cells after GSP-2 treatment

The cytokine productions of GSP-2 treated-human peripheral blood mononuclear cells (PBMCs) and monocyte-derived dendritic cells (moDCs) were assessed to evaluate the immunomodulatory activities of GSP-2. The PBMCs were isolated from fresh human buffy coat by density centrifugation using Ficoll-Paque™ Plus (GE Healthcare) as previously reported 7. For generation of moDC, monocytes (2×106 cells/mL) were first selected from PBMC by attachment on culture flask at 37°C, 5% CO2 for 45 min. The non-adherent cells were removed by washing 2 to 3 times with a gentle stream of medium. The adherent cells were cultured at 1×106 cells/mL in RPMI-1640 medium plus 10% (v/v) FBS supplemented with 50 ng/mL GM-CSF and 40 ng/mL IL-4. Immature dendritic cells (DC) were harvested after 6 days, and maturation was induced for 2 days by adding 10 ng/mL lipopolysaccharide (LPS) in culture medium. The cells were incubated at 37°C in a humidified atmosphere of 5% CO2.

The isolated human PBMCs and moDCs (2×10^6^ cells/ml) were then seeded in 96-well flat bottom microplates and incubated with GSP-2 from 0.3–1000 ng/ml. Lipopolysaccharide (LPS) (8 ng/ml) and a beta-glucan from *Euglena gracilis* (Sigma) (0.3–1000 ng/ml) were used as positive controls. After incubations of 18 h (for PBMCs) or 48 h (for moDCs) with the polysaccharides, the cell free supernatants were collected for cytokine ELISA experiments. The cytokines concentrations of IL-10 and IL-12 (from both PBMCs and moDCs) and IL-1, TNF-α (from PBMCs) were determined by ELISA kits according to the manufacturer instruction with detection limits ranged from 3.1 to 7.8 pg/ml.

### Statistical Analysis

All the experiments described here were repeated at least 2 times. Results are presented as mean ± standard error of the mean (SEM). Comparison of the data was performed using the one way analysis of variance (ANOVA) followed by Bonferroni post-test, as appropriate, were performed using GraphPad PRISM software version 5.0 (GraphPad Software, USA). Significance was defined as a * P value of <0.05, ** P<0.01, *** P<0.001.

## Result and Discussion

### The structure elucidation

**Figure 1:**
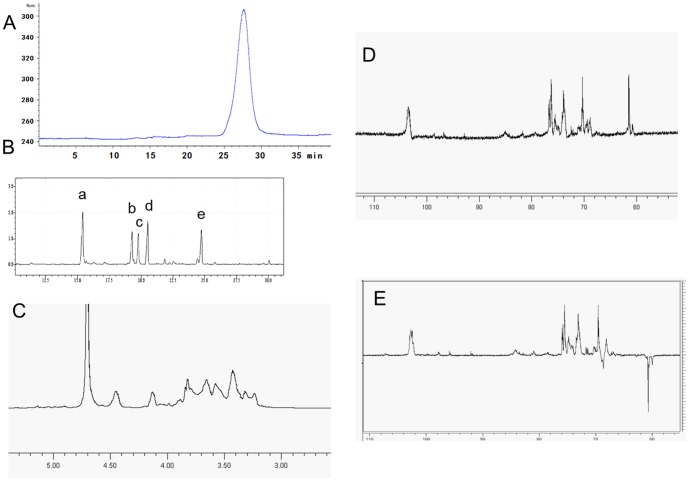
HPLC profile, methylation analysis result and 1D NMR spectrums of GSP-2. A: HPLC profile of GSP-2. Samples (2 mg/mL, 10 µL) were analyzed on an Agilent 1100 system equipped with an ELSD detector and TSK GMPW_XL_ gel filtration columns (7.8×300 mm×2), with 20 mM CH_3_COONH_4_ as mobile phase at 0.6 mL/min and column temperature at 40°C. Commercially available T-series dextrans (MW 2000, 670, 410, 270, 150, 80, 50, 12, 5 and 1 kD). B: Methylation analysis result of GSP-2. GC-MS tests for methylation analysis were measured with a DB-5 column (30 m×0.25 mm×0.25 µm), and at temperatures programmed from 170–225 °C at 2 °C/min and then hold on 10 min, in the figure, a: *T*-Glc*p*, b: *1,3*-linked Glc*p*, c: *1,4*-linked Glc*p*, d: *1,6*-linked Glc*p*, e: *1,3,6*-linked Glc*p*. C-E: 1D NMR (^1^H, ^13^C, DEPT) spectra of GSP-2 in D_2_O with TMS as external standard, obtained on a Bruker AM 500 spectrometer with a dual probe in the FT mode at room temperature.

GSP-2, with high homogeneity as indicated by the single and symmetrical peak in the HPLC-GPC-ELSD analysis shown in Fig. 1A, has an apparent molecular weight of 32 kDa as calculated from the calibrated standard curve. Sugar composition analysis indicated that GSP-2 was mainly composed of glucose as its sugar constituents, and absolute configuration study further revealed that all the glucose residues in GSP-2 are of D configuration. Besides, amino acid composition analysis revealed that 5.2% of this fraction is protein, which was composed of 16 kinds of amino acids as shown in the Table 1. Since the de-protein process has been repeated for ten times, it is supposed that these amino acids mainly remained on the sugar chain of GSP-2 by covalent bonds. The characterization will make this fraction very soft and flexible and facilitate to be recognized by the polysaccharide receptors on the surface of the immune cells, such as Toll-like receptor 4 on the surface of dentritic cells [23]–[24].

**Table 1:** Amino acid composition of the polysaccharide (GSP-2) isolated from the fruiting bodies of *Ganoderma sinense* ^a^.

Amino acid	Contnet µg/mg
Aspartic acid	2.73
Threonine	10.72
Serine	8.71
Glutamic acid	3.79
Glycine	3.00
Alanine	6.50
Vakine	3.38
Methiomine	0.56
Isoleucine	1.18
Leucine	2.55
Phenylalanine	3.09
Lysine	0.61
Histidine	0.71
Arginine	0.30
Proline	3.28
Tyrosine	0.55

a All the result were tested by a HITACHI automatic amino acid analyzer.

Methylation analysis revealed the exsitence of five types of sugar residues in GSP-2, *i.e.*, non-reducing terminal glucopyranosyl (Residue A, 27%), (1→3)–linked glucopyranosyl (Residue B, 16%), (1→4)–linked glucopyranosyl (Residue C, 15%), (1→6)–linked glucopyranosyl (Residue D, 22%), (1→3, 6)–linked glucopyranosyl (Residue E, 20%), (Fig. 1B, Table 2). In the FT-IR spectrum of GSP-2, the strong band at 3412.2 cm^−1^ was attributed to the hydroxyl stretching vibration of the polysaccharide, and that at 2917.27 cm^−1^ was due to the C–H stretching vibration absorption. Characteristically, the bands at 1000–1100 cm^−1^ suggested the presence of pyranose form of the glucosyl residue in the GSP-2 (Data not shown).

**Table 2:** GC–MS test result for the methylated sugar moieties of the polysaccharide GSP-2 ^a^ ^,^ ^b^.

Residue No.	Methylated sugars	Type of linkage	Molar ratio
A	2,3,4,6-Me4-Glc	Terminal Glc*p*	27%
B	2,4,6-Me3- Glc	1,3-Linked Glc*p*	16%
C	2,3,6-Me3-Glc	1,4-Linked Glc*p*	15%
D	2,3,4Me3- Glc	1,6-Linked Glc*p*	22%
E	2,4-Me2-Glc	1,3,6-Linked Glc*p*	20%

a The result were tested on DB-5 GC-MS column.

b All the sugar residues were primarily identified by their MS spectrum and further confirmed by their relative retention time to 2,3,4,6-Me4-Glc.

As the first step to understand the sequence of the sugar residues existing in GSP-2, a partial acidic hydrolysis was conducted and produced a degraded polysaccharide GSP-2-P with a yield of 63.5%. Methylation analysis by GC-MS revealed that GSP-2-P, with a molecular weight of 25 kDa, was mainly composed of the (1→6)–linked glucopyranosyl and (1→4)–linked glucopyranosyl sugar residues. Compared with the original polysaccharide, the increased molar ratio of *1,6*-linked Glc*p* and *1,4*-linked Glc*p* indicated that these two residues are existing in the backbone of GSP-2. In the meanwhile, the significant decrease of the molar ratio of *1,3,6*-linked-Glc*p* and *1,3*-linked Glc*p* revealed that *1,3*-linked Glc*p* is located on the side chain position, the backbone of GSP-2 has branches at *O*-3 (not *O*-6) position of *1,3,6*- linked-Glc*p.*

### NMR analysis

In the ^1^H NMR spectrum of GSP-2, due to the overlap of the D_2_O (*δ* 4.70-4.78), only one group of anomeric proton signals can be identified at *δ* 4.40*-*4.45. In the high field region, a group of sugar ring proton signals are observed at the field of *δ* 3.00 to *δ* 4.30 (Fig. 1C). In the ^13^C NMR spectrum of GSP-2, only one group of irresoluble anomeric signals are shown at *δ*103.3, in the high field region, a group of sugar ring carbon signals are shown in the field of *δ* 60.0 to *δ* 85.0 (Fig. 1D, 1E). As shown in^ 1^H and ^13^C NMR spectra of all the five glucopyranosyl residues (Res A-E), the appearance of the anomeric carbon signals at δ 103.3 and proton signals ranged from δ 4.45 to δ4.72 revealed that all the residues are of β anomeric configuration [25]-[26].

**Figure 2:**
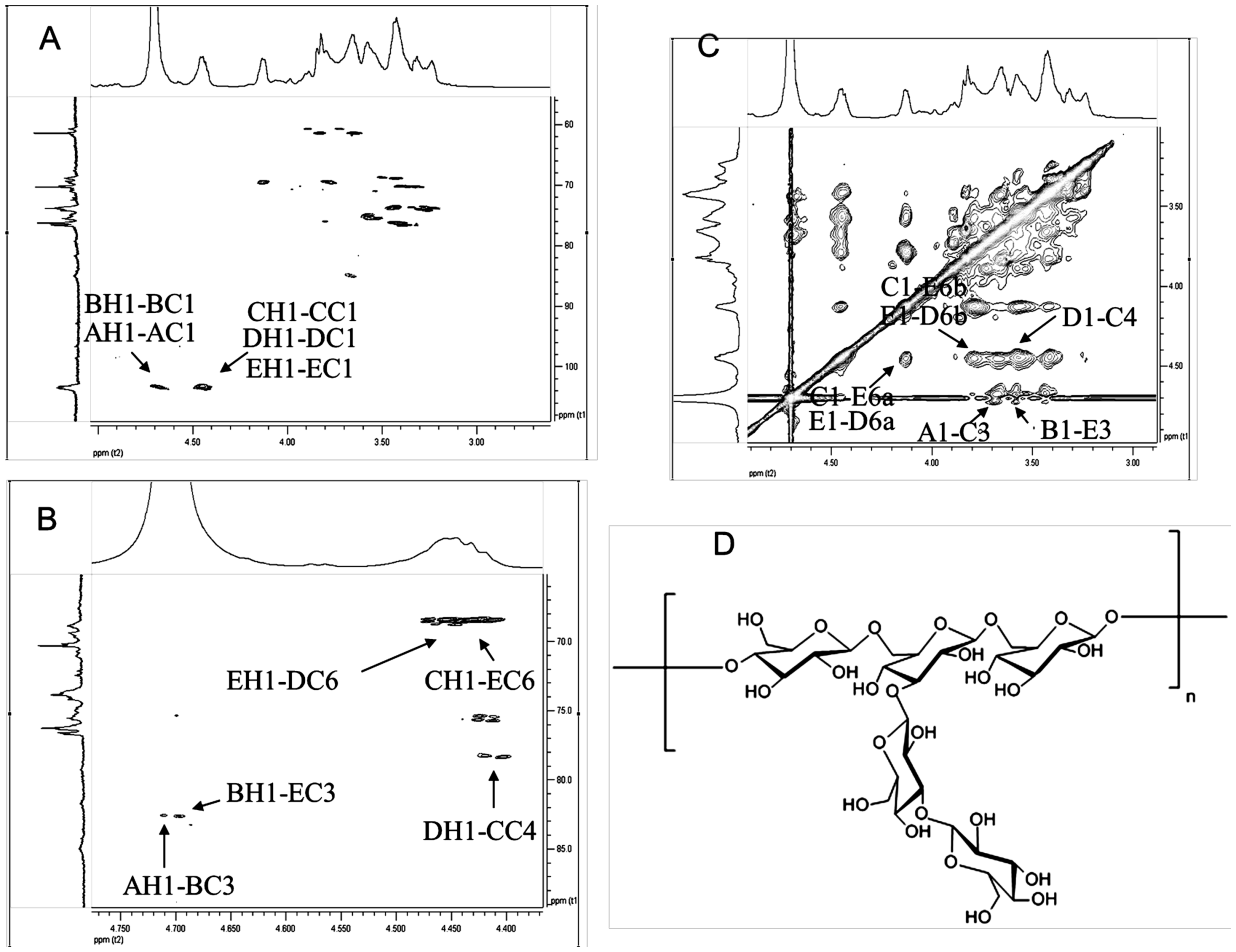
2D NMR spectrums and proposed structure of the polysaccharides from the fruiting bodies of *G. sinense*. (A: HSQC spectrum, B: HMBC spectrum, C: NOESY spectrum, D proposed strcuture).

To attribute the ^1^H and ^13^C NMR signals of GSP-2, a HSQC technique was employed to identify the correlation between the proton and carbon signals. From the spectrum, two correlations can be found in the anomeric region of the HSQC spectrum, *ie: δ* 4.70-4.72 (^1^H)*/δ* 103.3 (^13^C) and *δ* 4.40-4.45 (^1^H)*/δ* 103.3 (^13^C), as showed in the Fig. 2A, these two correlation signals can be employed as the start point to completely assigned all the ^1^H and ^13^C signals of GSP-2. Based on the 2D NMR data (H-H COSY, NOESY and HMBC), as listed in the Table 3, it can be deduced that the C-3 of residues B and E**,** C-4 of residue C were downfield to *δ* 84.4 and *δ* 78.5 due to the α effect of glycosylation, for the same reason, the C-6 signals of residues D and E were downfield to *δ* 69.2, which also can be confirmed by the reversed signal in the DEPT spectrum of GSP-2 (Fig. 1E) [27]-[28].

**Table 3:** ^1^H NMR and ^13^C NMR-chemical shifts of GSP-2 ^a^ ^,^ ^b^.

Glycosyl residue	H-1/C-1	H-2/C-2	H-3/C-3	H-4/C-4	H-5/C-5	H-6a,H-6b/C-6
^**β-D-Glc*p*-(1→**^	4.72	3.22	3.43	3.44	3.40	3.62, 3.82
A	103.3	73.8	75.8	70.2	76.0	60.9
^**→3)-β-D- Glc*p-(1→***^	4.70	3.42	3.65	3.35	3.38	3.62, 3.82
B	103.3	73.8	84.4	70.0	76.3	60.9
^**→4)-β-D -Glc*p-*(1→**^	4.42	3.22	3.36	3.58	3.60	3.61, 3.80
C	103.3	74.0	73.9	78.5	75.1	60.9
^**→6)-β-D-Glc*p*-(1→**^	4.41	3.22	3.43	3.44	3.58	3.77, 4.12
D	103.3	73.8	76.0	68.8	75.8	69.2
^**→3,6)-β-D-Glc*p*-(1→**^	4.45	3.48	3.65	3.43	3.58	3.77, 4.12
E	103.3	73.9	84.4	68.8	76.0	69.2

a Measured in 500/125 MHz, *δ* in ppm.

b Values of ^13^C chemical shift were record with reference to TMS as internal standard.

In this study, the specific linkages of glycosyl residues were further determined by the HSQC, HMBC and NOESY (Fig. 2A-C) experiments. The inter-residue HMBC correlations from H-1 of *Residue* ***C*** **(** ***C*** **)** to C-6 of *Residue* ***E*** **(** ***E*** **)**, and H-6 of *Residue* ***E*** to C-1 of *Residue* ***C***, in collaboration with the inter-residue NOESY effects of H-1 (***C***) with H-6 (***E***), established the linkage of residues C-1 of *Residue* ***C*** to *O-6* position of *Residue* ***E***. The inter-residue HMBC correlations from H-1 of *Residue* ***E*** to C-6 of *Residue* ***D***, and the H-6 of *Residue* ***D*** to C-1 of *Residue* ***E***, in collaboration with the inter-residue NOESY effects of H-1 (***E***) with H-6 (***D***), established the linkage of residues C-1 of *Residue* ***E*** to *O-6* position of *Residue* ***D***. The inter-residue HMBC correlations from H-1 of *Residue* ***D*** to C-4 of *Residue* ***C***, and the H-4 of *Residue* ***C*** to C-1 of *Residue* ***D***, in collaboration with the inter-residue NOESY effects of H-1(***D***) with H-4 (***C***), established the linkage of residues C-1 of *Residue* ***D*** to *O-4* position of *Residue* ***C***. The observed HMBCs from H-1 (***B***) to C-3 (***E***), together with NOESY effect of H-1 (***B***) with H-3 (***E***), constructed the branch substitution located at C-3 of *Residue* ***E***. Similarly, the HMBCs from H-1 (***A***) to C-3 (***B***), in combination with the corresponding inter-residue NOESY effects of H-1 (***A***) with H-3 (***B***), revealed that the terminal *Residue* ***A*** attached at C-3 of *Residue* ***B.***

After comprehensive composition analysis, methylation analysis and NMR experiments, it thus can be concluded that GSP-2 is a protein-bound polysaccharide having a backbone composed of (1→4)- and (1→6)-linked β-D-glucopyranosyl residues, bearing the side chains composed of (1→3)- and terminal-linked β-D-glucopyranosyl residues at *O*-3 position of (1→6)-linked β-D-glucopyranosyl residues in the backbone (Fig. 2D).

### Immunomodulatory effect

**Figure 3:**
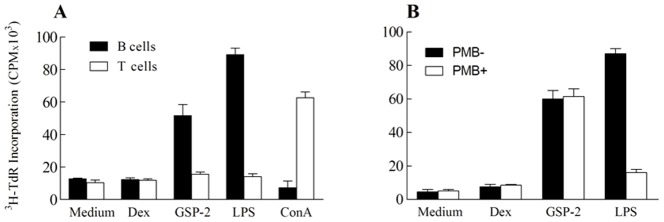
Stimulating effect of GSP-2 on the proliferation of the mouse splenocytic B and T cells. Freshly fractionated splenocyte, splenic B (solid bars) and T (open bars) cells were stimulated with, or without (*Medium*), dextran or GSP (30 µg/ml). LPS (2 µg/ml) and ConA (2 µg/ml) were included as controls. In a parallel experiment, mouse splenocytes were stimulated with, or without (*Medium*), GSP (30 µg/ml), dextran (30 µg/ml) or LPS (10 µg/ml) in triplicate wells in the presence, or absence, of PMB. ^3^H-TdR was added to the cultures for the last 8 hrs of incubation and then ^3^H-TdR incorporation (CPM) of each well counted. A: Parallel experiment to exclude the influence of the endotoxin contamination; B: Stimulating effect of GSP-2 to the mouse splenocytic B and T cells. All results are presented as mean ± SEM, *, P<0.05; **, P<0.01; ***, P<0.001 for difference from culture without treatment. (n = 9, repeated 3 times).

As shown in^ 3^H thymidine incorporation assay, GSP-2 significantly stimulated the proliferation of the mouse splenocyte in a dose-dependent manner from 10 to100 µg/mL for 48 h. The influence induced by the accidently contamination of the endotoxin was excluded by adding a specific inhibitor of lipopolysaccharide (Polymixin B, PMB, 10 µg/mL) to the medium (Fig. 3A). Further study on isolated cells indicated that both GSP-2 (30 µg/mL) and LPS (2 µg/mL) induced vigorous proliferation of mouse splenic B cells, while GSP-2 showed no effect on splenic T cells (Fig. 3B).

**Figure 4:**
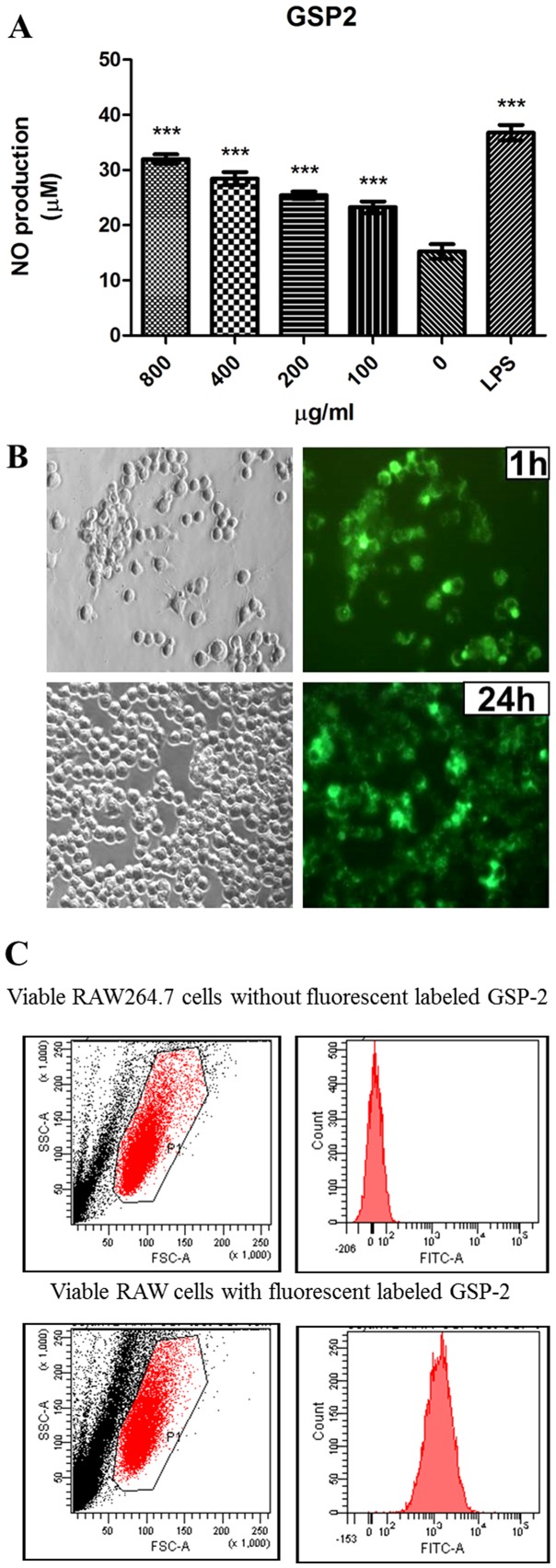
Nitric oxide production and phagocytosis of GSP-2-treated RAW 264.7 cells. A: The levels of NO production of RAW264.7 cells were assessed after incubation with GSP-2 or LPS for 24 h using Griess assay. All data are expressed as mean ± SEM of three individual experiments (n = 12). Significant difference: *, P<0.05; **, P<0.01; ***, P<0.001 for difference from culture without treatment. B: The viable RAW 264.7 cell populations were gated (left) and the FITC-positive population was shown in fluorescent-GSP-2-labeled cells (lower right histogram). C: RAW 264.7 cells were incubated with 10 µg GSP-2 (fluro-labeled) for 24 h. The cells were imaged at the 1^st^ and 24^th^ hour (lower histograms). After 24 h incubation, the cells were collected, washed and resuspended in PBS and the fluorescence of the samples was detected by flow cytometry. (Upper histograms: viable RAW 264.7 cells without fluorescent labeled GSP-2, lower histograms, 1^st^ hour: viable RAW 264.7 cells with fluorescent labeled GSP-2, 24^th^ hour).

The immunomodulatory activities GSP-2 were also evaluated in mouse macrophage like cell line (RAW 264.7). As shown in Fig. 4A, GSP-2 significantly stimulated the production of nitric oxide (NO) in RAW 264.7 cells in a dose-dependent manner. As well known that NO is an important chemokine secreted by macrophage that can oxidize the pathogens, the secretion of NO is related to the swallowing of the cells to the pathogens, such as bacteria and virus [29] Some biological macromolecules, such as polysaccharides also can be identified as non-self and phagocytosed by macrophages, and the cell will further degrade the polysaccharides to small deribs and present to the other immune cells. In order to investigate the phagocytosis of the macrophages to our polysaccharide fraction, a fluorescent tag was specifically labeled on the reducing end of polysaccharide GSP-2 backbones, and then added to the macrophage culture medium. The cells were visualized and imaged under inverted fluorescent microscope after 1 h and 24 h (Fig. 4B). The number of fluorescent-labeled cells was increased after 24 h incubation with labeled GSP-2. The fluorescence of the labeled and unlabeled cell samples was also detected by flow cytometry as shown in Fig. 4C. These results demonstrated that polysaccharide GSP-2 could be phagocytosed by RAW 264.7 cells, in which biological response (such as NO production) was induced.

It is suggested that GSP-2 is a typical B cell and macrophage dependent immune-modulator. Polysaccharides from medicinal materials could exhibit different immunopotentiating activities. For example, polysaccharides found in the roots of *Acanthopanax koreanum*, *Acanthopanax senticosus*, *Platyloden grandiflorum* and *angelan* are also able to activate mouse B cells and macrophages but not T cells B cells [30]-[31]. Polysaccharides from *P. linteus* can stimulate B cells, T cells and macrophages [32], while lentinan and β(1→3)-glucan isolated from *Lentinus edodes* is a stimulator of T cells and macrophages, but not B cells [33]. The relationship between polysaccharide origin, structure and their immuno-modulation activity remains to be further characterized.

**Figure 5:**
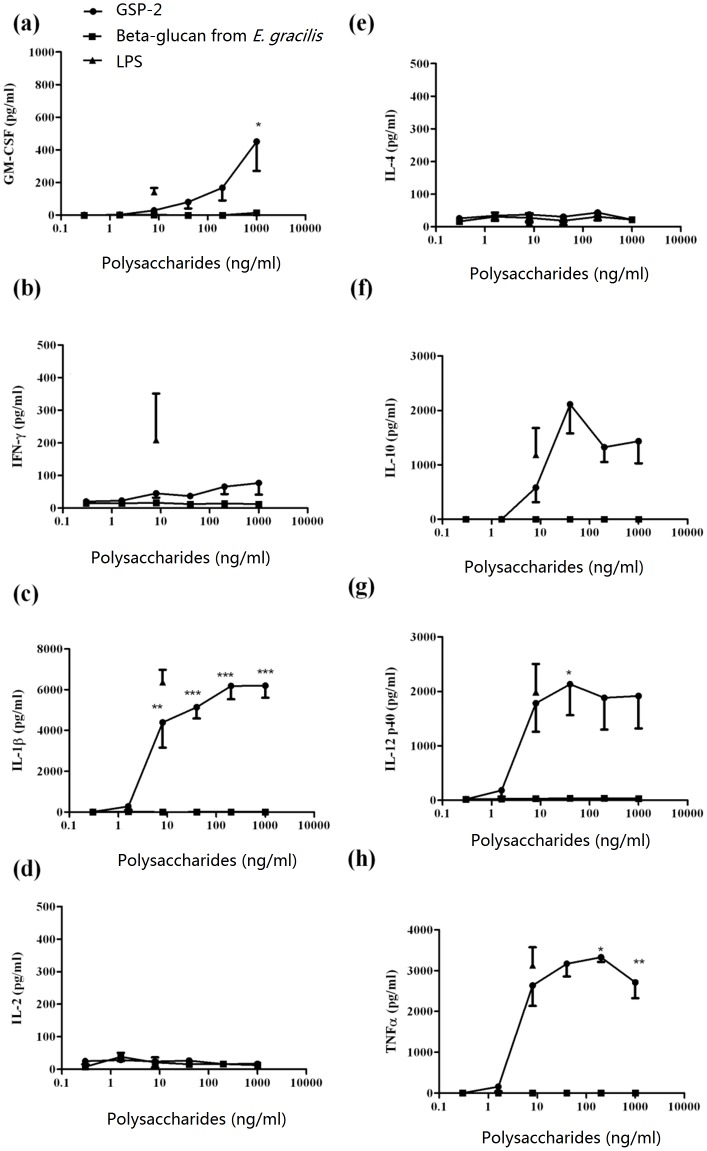
Cytokine productions of GSP-2-treated PBMCs. Culture supernatants were collected 24-2 and the cytokines concentrations were specifically determined by ELISA. All data are expressed as mean ± SEM of three individual experiments (n = 12). Differences between the treated and untreated control groups were compared using one-way ANOVA. * P<0.05, ** P<0.01, *** P<0.001 for difference from culture without treatment.

**Figure 6:**
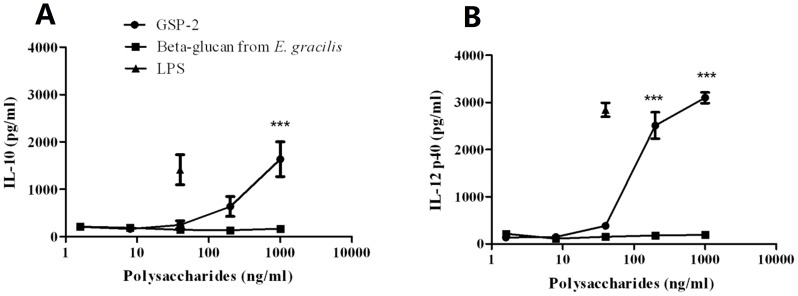
Cytokine productions of GSP-2-treated moDC. Culture supernatants were collected 48-2 and the cytokines concentrations were specifically determined by ELISA. Lines represented mean percentage ± S.E.M. of duplicates (n = 8). LPS and Beta-glucan from *E. gracilis* were used as positive control. Significant difference: *, P<0.05; **, P<0.01; ***, P<0.001 for difference from culture without treatment.

Regarding the receptor of these immune-activity polysaccharides on B cells and macrophages, some have been generally acknowledged, like mannose receptor, CR3 (CD11b/CD18), lactosylceramide, dectin-1, Toll-like receptors (TLR) and so on. Polysaccharides from the roots of *Acanthopanax koreanum* and *Platyloden grandiflorum* stimulate B cells through TLR2, TLR4 and also CD19 and CD79b [31], [34]. Ando *et al* reported that safflower polysaccharides activated NF-κB signaling pathway via TLR4 in macrophages [35]. Polysaccharides may employ molecules such as CD14, RP105, MD-1 or MD2 as a bridge to indirectly interact with TLR4. Ongoing experiments in our laboratory will search for potential receptor of GSP-2 on B cells and macrophages. Apart from the immuno-stimulating effects of GSP-2 in mouse immune cells, the immunomodulatory activities of GSP-2 in human PBMCs and moDCs were also elucidated so that the clinical potential of the polysaccharide could be revealed. As shown in Fig. 5, the productions of TNF-α, IL-1β, IL-12 and GM-CSF were increased dose-dependently in GSP-2-treated PBMCs. Besides, GSP-2 could also stimulate the IL-10 and IL-12 productions of human monocyte-derived dendritic cells in a dose dependent manner (Fig. 6). Dendritic cells (DCs) are the antigen presenting cells that initiate and direct adaptive immune responses, capable of inducing protective adaptive immune responses [36]. And IL-10 and IL-12 are two important mediators secreted by DCs: IL-10 is a pleiotropic cytokine with anti-inflammatory and immunosuppressive properties [37], which can act as a feedback regulatory role to constrict the immune response when DC is activated [38]; And IL-12 can polarizes the immune system toward a primary Th1 cell response [38]–[39]. These result indicated that GSP-2 could robust the response of the dentritic cells to the pathogens.

It is know that the beta glucan from different source might own different structure. Beta-glucan from oat are of a linear mainly structure of 1,4-*β*-Glc*p* residues, while mushroom sourced beta glucan often bear a backbone structure of 1,3-*β*-Glc*p* residues or 1,6-*β*-Glc*p* residues, and branched by the side chain of 1,3-*β*-Glc*p* residues. In this study, GSP-2 has a very novel backbone structure of 1,6-, 1,4-*β*-Glc*p* residues. As GSP-2 is highly water-soluble, we suppose that it might share some similar characters with the well studied yeast water-soluble beta-glucan (PGG), which could be identified by the toll-like receptor-4 on the surface of the cells. The influence of the structural factors such as the length of the backbone, degree of polymerization, degree of branching and the length of the side chain to the interaction between this fraction and the toll-like receptors deserve further studies [40].

In summary, a water-soluble protein-bound glucan GSP-2 was isolated from the medical mushroom *G. sinense,* with an apparent molecular weight of 32 kDa. Structural analysis revealed that GSP-2 contains a backbone composed of (1→4)- and (1→6)-linked β-D-glucopyranosyl residues, bearing the side chains of (1→3)- and terminal-linked β-D-glucopyranosyl residues at *O*-3 position of (1→6)-linked β-D-glucopyranosyl residues in the backbone. In addition, GSP-2 was found to be a B cell dependent immunomodulator without any *in vitro* stimulating effect to the mouse splenetic T cells, and could significantly stimulating cytokine secretion from PBMCs and derived dendritic cells. More interestingly, the fluorescent labeled GSP-2 could be phagocytosis by mouse macrophage like RAW264.7 cells, in the meanwhile, enhanced the nitric oxide production of the cells, suggesting that the endocytosis function of the innate immune cells might partially explain the absorption of protein-bound polysaccharides.

## Supporting Information

Checklist S1 ARRIVE Checklist.
(DOC)
